# Total Mandibular Subapical Alveolar Osteotomy to Correct Class II Division I Dentofacial Deformity

**DOI:** 10.1155/2018/5469481

**Published:** 2018-10-03

**Authors:** Rafael Correia Cavalcante, Isabela Polesi Bergamaschi, Aline Monise Sebastiani, Fabiano Galina, Marina Fanderuff, Delson João da Costa, Nelson Luis Barbosa Rebellato, Rafaela Scariot, Leandro Eduardo Klüppel

**Affiliations:** ^1^Oral and Maxillofacial Surgery Department, Federal University of Parana, Curitiba, Brazil; ^2^School of Health Science, Positivo University, Curitiba, Brazil

## Abstract

**Introduction:**

Class II division I malocclusions are the most common dentofacial deformities seen in clinical practice. Severe cases or cases in which growth has ceased may require full correction combining orthodontic and surgical treatment. We report a case of a total mandibular subapical alveolar osteotomy, performed to correct a class II division I dentofacial deformity.

**Case Report:**

A 19-year-old female patient was referred to the oral and maxillofacial surgery department at the Federal University of Paraná with chin aesthetic complaints as well as class II malocclusion. The proposed treatment was total mandibular subapical alveolar osteotomy, retaining the chin position and eliminating the need for genioplasty, since, although the patient presented with a class II dentofacial deformity, the chin was well positioned. Under general anesthesia, a “V-shaped” incision was conducted from the right retromolar region to the left retromolar region. A ring of cortical bone was removed around the mental foramen, with the aim to create a space around the mental nerve. Fixation was conducted with plates and screws of the 2.0 system. The patient on six-year follow-up showed osteotomy stability, a better overall occlusion, and outcome satisfaction.

## 1. Introduction

Class II division I malocclusions are the most common dentofacial deformities seen in clinical practice and are reported to occur in 13% of the population. Combined orthodontic and surgical treatment in cases of severe class II dentofacial deformities is a routine procedure in these orthodontic patients. Mild to moderate class II dentofacial deformities in young patients (whose growth has not ceased) can only be managed with orthodontic treatment [1]. Moderate or severe mandibular retrusion cases, however, are commonly treated with bilateral mandibular ramus sagittal split osteotomy (BSSO). Depending on the nature of the problem and its severity, surgical correction of class II division I malocclusion can involve one or both jaws [2, 3].

The specific mandibular osteotomy technique to correct this type of deformity was first described by Hofer in 1942 [4]; however, surgery was limited to the anterior mandibular region alone. Hofer's original technique was modified and popularized by Kole only in 1959 [3]. The total mandibular subapical alveolar osteotomy (TMAO) was developed by MacIntosh in 1974 [4], mainly for the correction of anterior open bite. Dietz et al. [5] and Murray [6] reported further modifications to the total mandibular osteotomy technique, which included a horizontal and medial ramus cut above the lingula. The main indications of TMAO are as follows: class II division I incisor relationship, particularly in cases where the chin is well positioned at the beginning of the treatment [1, 7], pogonion kept in the same position to increase the lower facial height [8]; class II division II low-angle malocclusion uncorrected by orthodontic treatment alone, especially in patients that need advancement but have an excessive mental projection [9]; mandibular vertical alveolar deficiency; anterior open bite; mandibular ramus sagittal split osteotomy relapse; cases of condylar agenesis and hypoplasia; lateral open bite [4, 10]; and in cases that need a profound change in the mentolabial sulcus [1, 11].

Pangrazio-Kulbersh et al. [11] compared the stability between the total mandibular subapical osteotomy and bilateral sagittal split ramus osteotomy (BSSO) for correction of class II subjects. Stability yielded long-term results with both techniques. Mandibular advancement with the use of BSSO associated with rigid fixation has been shown to improve stability by resisting slippage at the osteotomy site because of the stretch of the paramandibular connective tissues, suggesting high stability. A reduction in relapse is also observed with TMAO because the suprahyoid muscles are not disturbed during the alveolar segment advancement. The authors suggest, however, that total TMAO is the procedure of choice in those cases where a profound change in labiomental sulcus is needed. Changes in the profile that resulted from TMAO appear to be more satisfactory than those which resulted from BSSO associated with genioplasty. A reduction of chin could yield a squarer mandible in patients who are already brachycephalic with increased bigonial width. The other advantage is the possibility of anterior, vertical, and posterior repositioning of the mobilized mandibular alveolar segment [12]. TMAO is a meticulous procedure that requires time and care with mental nerves. In addition to the risk of paresthesia, it has a risk of loss of pulp vitality and one or more teeth or even the entire dentoalveolar segment [4]. According to Scheideman et al. [13], Zisser and Gattinger [14], and Allen et al. [15], TMAO has no long-term deleterious effects on pulp vitality nor the alveolar segment.

The mandibular technique more commonly used to correct mandibular deficiencies is BSSO because it is a versatile and easy technique. However, other surgical techniques must be considered under certain conditions and in specific clinical cases. Although TMAO is an alternative for correction of mandibular deficiency, only a few cases have been reported in literature. The aim of the present study is to report a case of class II division I dentofacial deformity with good projection of chin, corrected through total subapical mandibular osteotomy.

## 2. Case Report

**Figure 1:**
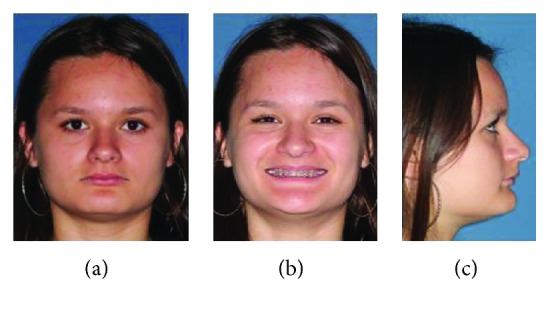
(a–c) Preoperative facial images showed a well-positioned maxilla and favorable chin projection associated with a deep labiomental sulcus.

**Figure 2:**
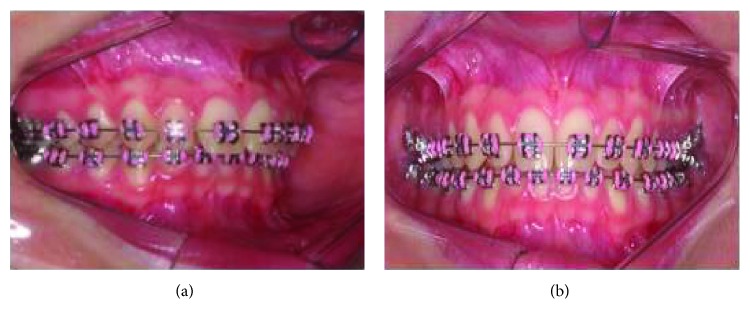
(a, b) Preoperative intraoral images showing a deep bite and retrusion of the inferior alveolar segment.

**Figure 3:**
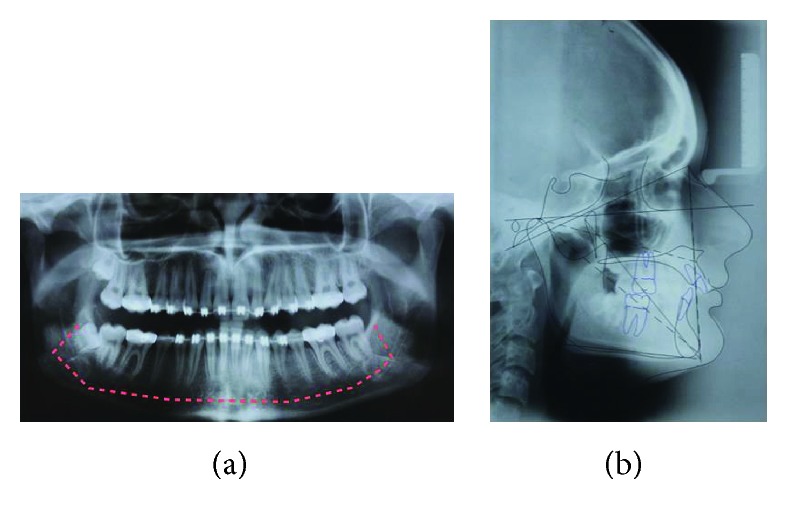
(a) Preoperative panoramic radiography with subapical osteotomy simulation. (b). Digital cephalometric tracing of preoperative lateral radiograph using Dolphin Imaging Software.

A 19-year-old, white female patient was referred to the oral and maxillofacial surgery department at the Federal University of Paraná as she complained regarding her aesthetics and malocclusion. Facial analysis showed a well-positioned maxilla despite a hypodivergent face pattern, with a reduction of tooth exposure upon smiling, and favorable chin projection associated with accentuated and deep labiomental fold due to retrusion of the inferior alveolar segment (Figures 1 and 2). There was also a shortening of the lower third of the face. There was no transversal deformity. Preoperative imaging exams showed a favorable position of the maxilla associated with good inclination of maxillary and mandibular incisors. A class II malocclusion with a deep bite in association with accentuated curve of Spee (COS) was found. The chin (pogonion) was well positioned (Figure 3). The lower third molar was extracted six months before the time of orthognathic surgery. Different treatment options to correct the mandibular retrusion were offered to the patient: bilateral sagittal split ramus osteotomy (BSSO) associated with backward genioplasty or total subapical mandibular osteotomy (TMAO) which would keep the chin in position and eliminate the need for genioplasty. Another option was combined orthognathic surgery on the mandible with BSSO or TMAO, associated with forward and downward repositioning of the maxilla. The patient chose the total subapical mandibular osteotomy procedure only.

**Figure 4:**
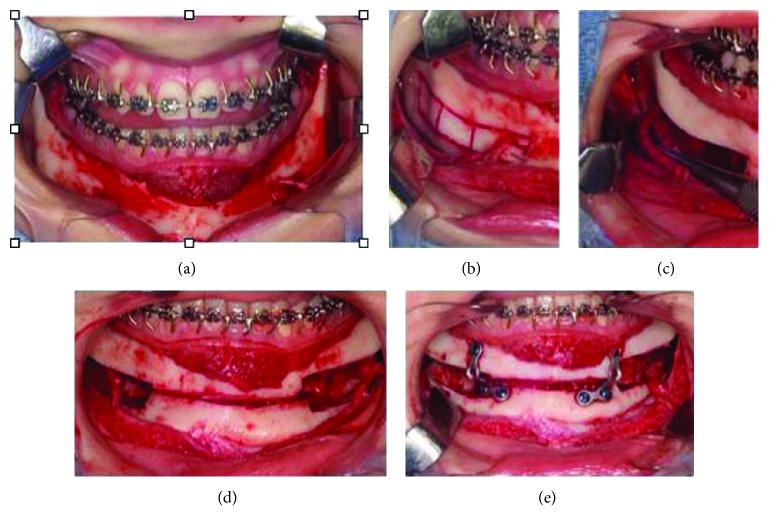
Surgical technique. (a) A “V” incision was conducted from the right to left retromolar region, and a mucoperiosteal flap was carefully detached to maintain the mental nerve integrity. (b) A ring of cortical bone was removed around the mental foramen, aiming to create a space around it. A gradual and careful removal of the buccal cortical bone with a drill exposed the inferior alveolar neurovascular bundle and let it free in all of its extension from the foramen to the retromolar region. (c) Once the inferior alveolar neurovascular bundle was viewed in its entirety, it was carefully removed from the inferior dental canal and repositioned either inferiorly or superiorly to it. (d) After nerve repositioning, a reciprocate saw was carefully used to conduct an osteotomy from the mental foramen to the retromolar region, to divide the lingual cortical bone from the basal bone, careful to avoid damage to the posterior teeth's apices. (e) After the dentoalveolar segment containing the entire lower dentition was mobilized, it was repositioned to the desired site and stabilized with miniplates and screws of the system 2.0.

**Figure 5:**
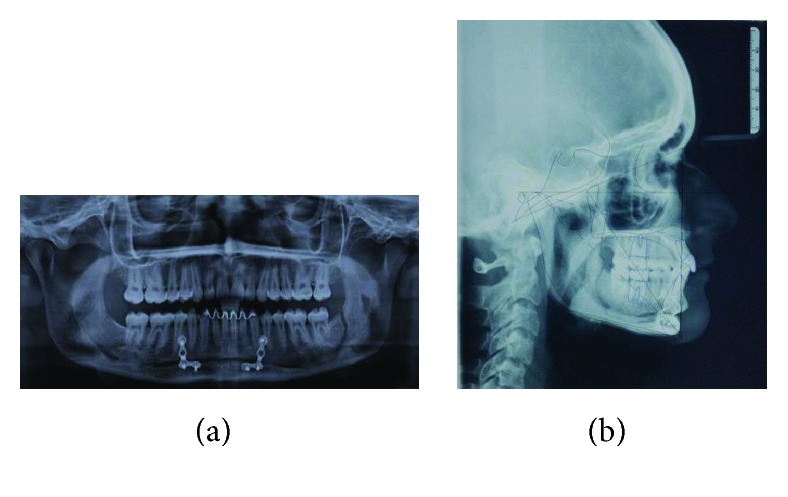
(a) Postoperative panoramic radiograph. (b) Digital cephalometric tracing of postoperative lateral radiograph using Dolphin Imaging Software.

After 39 months of orthodontic treatment, surgery was performed under general anesthesia. After a local bupivacaine 0.5% infiltration, a “V” incision was conducted from the right to the left retromolar region, and a mucoperiosteal flap was carefully detached to maintain the mental nerve integrity (Figure 4(a)). A ring of cortical bone was removed around the mental foramen, aiming to create a space around it. A gradual and careful removal of the buccal cortical bone with a drill, exposing the inferior alveolar neurovascular bundle and letting it free in all of its extension from the foramen to retromolar region, was done (Figure 4(b)), different from the original technique [5]. Once the inferior alveolar neurovascular bundle was viewed in its entirety, it was carefully removed from the inferior dental canal and repositioned either inferiorly or superiorly. After the repositioning, a reciprocate saw was carefully used to conduct an osteotomy from the mental foramen to the retromolar region to divide the lingual cortical bone from the basal bone, taking care to avoid damage to the posterior teeth's apices (Figure 4(c)). The posterior vertical cut on the posterior body area behind the second molar was also done with 5 mm to safety margin of the tooth. The osteotomy was conducted until the parasymphyseal region, through the buccal and lingual plates, approximately 5 mm below the apices of the anterior teeth, taking care not to damage them. It is crucial to avoid as much possible damage to the lingual mucosa when cutting the lingual cortical bone due to the fact that it is the main pedicle supplying nutrition to the dentoalveolar segment after the osteotomy is completed (Figure 4(d)). After the dentoalveolar segment containing the entire lower dentition was mobilized, it was repositioned to the desired site and stabilized with miniplates and screws of the system 2.0. The conducted incision was closed with absorbable sutures (Figure 4(e)). Panoramic radiograph and cephalometric radiograph showed occlusal stability as well as the condyle in the right position after surgery (Figure 5). Postoperative orthodontics consisted of intercuspidation and adjustment of the COS, since an intentional open bite in the bicuspid area was left during surgery to allow increase of the anterior facial height. The patient finished orthodontic treatment after 12 months. In Table 1, it is possible to observe the measurements obtained from the lateral radiograph, pre- and postoperatively. Improvement in the measurements related to overjet, facial axis, jaw angle, inferior sulcus to H-line, and occlusal plane is noteworthy.

**Figure 6:**
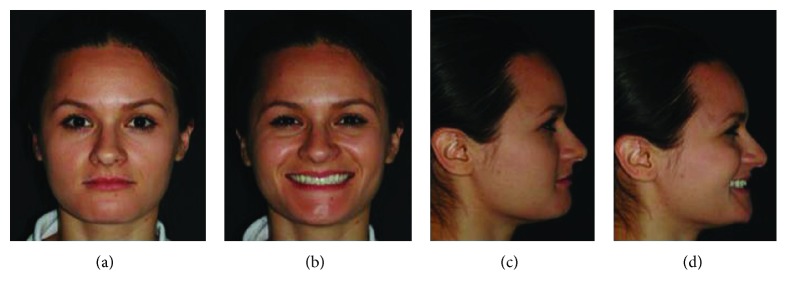
Correction of deep bite and dental retrusion associated with occlusal stability (6-year follow-up).

**Figure 7:**
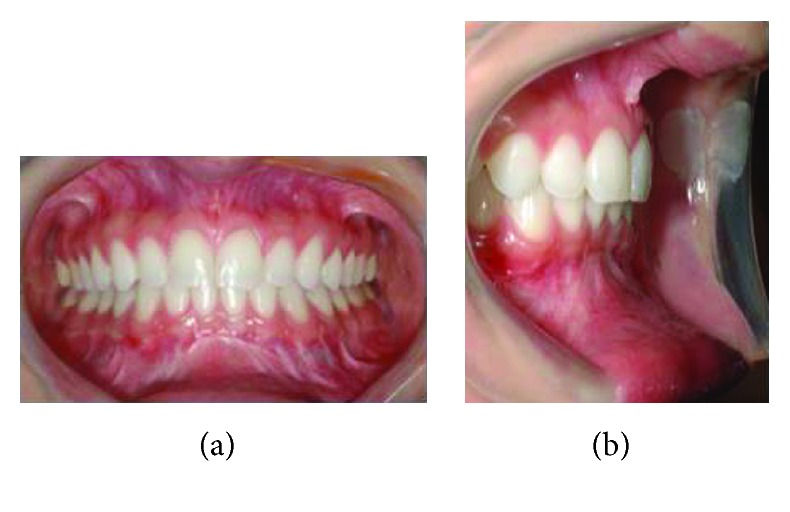
Occlusal stability associated with aesthetic face (6-year follow-up).

**Figure 8:**
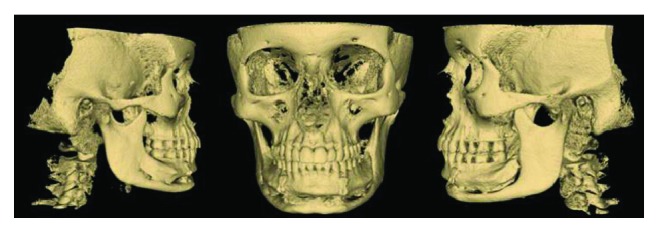
Reconstructed computerized tomography images showing bone deposition and stability of the osteotomized segments (6-year follow-up).

The six-year follow-up showed stability of the osteotomized segments with maintenance of plates and screws. Occlusal stability was also observed, associated with a 49 mm mouth opening. The labiomental sulcus was observed to be less deep in comparison to the preoperative stage. Patient reported of paresthesia in the mental region, which was expected, due to incisal nerve sectioning. The resolution of the main aesthetic complaint of the patient, which was a deep labiomental sulcus, was achieved through the TMAO (Figures 6, 7, and 8).

## 3. Discussion

Cases where total subapical mandibular osteotomy (TMAO) technique was used to correct class II patients are relatively few. The bilateral sagittal split ramus osteotomy (BSSO), on the other hand, is a widely used technique and has been an extensively described technique for mandibular retrusion correction [1]. Some of the reasons for this may be surgical complexity in bilateral dissection of inferior alveolar nerves, more probability for nerve lesions posttotal subapical mandibular osteotomy, and longer operative time when compared to the BSSO (1.5–2 times long) [1, 2, 16–18]. Boye et al. [1] stated that TMAO is a technique that allows mandibular advancement with excellent aesthetic and functional results in class II patients with good chin position. According to the author, patients subjected to BSSO had more postoperative pain and swelling than those subjected to TMAO, due to the fact that in the TMAO there is no fracture of the basal bone which favors postoperative repair. However, due to possible complications, this technique should only be performed by experienced surgeons and in cases with complete indications. In the present case report, the BSSO technique was not chosen due to the fact that the patient's soft tissue projection of chin was in good position; however, the dentoalveolar segment was retracted. As mentioned earlier, the use of this technique would result in an accentuated projection of the pogonion, so another retrusion/impaction genioplasty surgery would be necessary. Other option for the patient was the use of Kole modification osteotomy technique to obtain a better chin, but this technique is more used to correct anterior open bite, and there would be a need for bone graft if significant movement is planned [19]. In the present case report, the patient was able to open and close her mouth with little discomfort from the immediate postoperative period, and as mentioned earlier, swelling was less when compared to the traditional approach [1, 2, 6, 7].

There are a few recent studies with the use of TMAO. Pangrazio-Kulbersh et al. [11] compared the BSSO with TMAO in patients with skeletal and dental class II. Twenty patients with mandibular retrognathia were treated with BSSO, and the other twenty with dentoalveolar retrusion were treated with TMAO. Both techniques yielded long-term stability even though TMAO had better results in reducing the depth of the mentolabial sulcus. This finding has proven a clinical significance in using TMAO for those patients in whom an alteration in the mentolabial sulcus is desired. In deep bite cases, total subapical mandibular osteotomy also increased the lower facial height [7, 8, 20], just like the BSSO since the mandible rotates downwards and forwards. None of the hard tissue changes were significantly different between the 2 procedures, except for a large increase in anterior facial height observed in the BSSO group [1]. More anterior displacements of the mandibular incisors, second molars, and pogonion were also observed in the BSSO group. Eliades and Hegdvedt [21] treated a class II division II skeletal discrepancy by using a combination of BSSO and TMAO over the standard BSSO accompanied by the genioplasty technique. The authors suggested that the main advantage of BSSO-TMAO technique over BSSO-genioplasty combination is the relatively decreased advancement required, because the surgical movement is limited to the extent dictated by the maxilla-mandibular skeletal relationship, as opposed to the dental relationship (dental overjet). Mohammed-Ali et al. [10] proposed a modification in TMAO osteotomy involving a different technique of decortication of the inferior alveolar nerve. The authors suggested that this modification is efficient in reducing what otherwise would be a time-consuming process.

Drawbacks associated with the total mandibular subapical osteotomy include the following: the risk of inferior alveolar nerve and dental root damage and the fact that this procedure is technically laborious and time consuming. To minimize these risks, piezosurgery has proven to be a valid alternative to the use of drills or traditional instrumentation. It is helpful and safe for conducting different surgical procedures involving removal of a bone in close proximity to noble structures, such as nerves and blood vessels. Peripheral nerves exposed in direct contact with piezosurgery were not transected. Functional and structural damage related to the force applied on the nerve, rather than to the ultrasonic microvibrations, was observed [22]. Even though piezosurgery was not used in the case reported in this paper, the team highly recommends it. In our case report, post-TMAO, the patient reported of mild paresthesia in the mental region, which was expected, but not affecting her quality of life. She did not report of difficulty in masticatory function and phonation nor in personal relationships.

## 4. Conclusion

TMAO allowed the anteroposterior and vertical correction of the malocclusion and elimination of deep labiomental sulcus, and no complications in the dentoalveolar portion were observed. The authors believe that when fully indicated and well conducted, TMAO will result in excellent functional and aesthetic results, with stability of the osteotomized segments maintained with plates and screws. Occlusal stability was also observed, associated with a 49 mm mouth opening. Patient reported of paresthesia in the mental region, which was expected.

## Figures and Tables

**Table 1:** Values from the digital cephalometric tracing done in the lateral radiograph, comparing pre- and postoperative measurements, using Dolphin Imaging Software.

Cephalometric measurements	Preoperative values	Postoperative values	Norms
SNA (°)	85.5	86.2	82.0 ± 1.0
SNB (°)	80.1	84.7	80.9 ± 1.1
ANB (°)	5.4	1.5	1.6 ± 2.5
Overjet (mm)	7.0	2.2	2.5 ± 1.8
Overbite (mm)	3.5	3.4	2.5 ± 0.5
Lower face height (ANS-Xi-Pm) (°)	34.9	35.9	45 ± 2.5
FMA (MP-FH) (°)	9.8	13.8	23.9 ± 3.1
SN-GoGn (°)	13.0	17.6	32.9 ± 3.8
Facial axis-Ricketts (NaBa-PtGn) (°)	104.7	98.5	90.0 ± 4.2
Gonial/jaw angle (Ar-Go-Me) (°)	113.1	119.4	122.9 ± 1.5
Chin thickness (Pg-Pg′) (mm)	12.6	16.4	13.9 ± 0.4
Inferior Sulcus to H-Line (mm)	8.4	4.8	4.0 ± 2.2
Occlusal plane to SN (°)	5.0	10.0	14.4 ± 3.7
